# The properties and formation mechanism of oat β-glucan mixed gels with different molecular weight composition induced by high-pressure processing

**DOI:** 10.1371/journal.pone.0225208

**Published:** 2019-12-27

**Authors:** Rui Fan, Peihua Ma, Dan Zhou, Fang Yuan, Xueli Cao

**Affiliations:** 1 Beijing Advanced Innovation Center for Food Nutrition and Human Health, Beijing Technology & Business University, Beijing, P. R. China; 2 Department of Nutrition and Food Hygiene, School of Public Health, Peking University, Beijing, P. R. China; 3 College of Food Science and Nutritional Engineering, China Agricultural University, Beijing, P. R. China; 4 School of Life Science and Technology, Beijing University of Chemical Technology, Beijing, P. R. China; University of Hyderabad, INDIA

## Abstract

High pressure, an emerging nonthermal technology has been widely applied in food product modifications. The effects of oat β-glucan concentration and pressure on the properties of mixed gels with the different ratios of varying molecular weight (MW) β-gulcan induced by HPP were investigated. The results showed that the lowest β-glucan concentration forming a gel was 15% at 200 MPa, while 8% β-glucan was required to form a gel at 500 MPa. The gel intensity and textural properties increased with elevating β-glucan total concentration and pressure. The characteristic compact and smooth mixed gel formed with 12% β-glucan at a ratio of 50:50 at 400 MPa for 30 min. Under this optimal parameters, the mixed solution showed a relatively lower particle size and turbidity, and the hydrogen bonding and electrostatic interaction played the main role during the gel formation process by high pressure. In addition, the core molecular structure of β-glucan was maintained in the mixed gel formed under the optimal parameters.

## Introduction

US Food and Drug Administration (FDA) approved the health claim in 1997, which included that the intake of oat-based foods could decrease the risk of heart disease, and oat bran was registered as the top oat-based food in reducing cholesterol at a dosage of 3g β-glucan each day and a minimum of 0.75 g β-glucan in each serving [1]. It is well known that β-glucans in cereals, especially oats, reduce blood glucose [2] and serum cholesterol levels [3], regulate immunity [4] and improve antioxidant activity [5]. It was reported that the viscosity of β-glucan played an important role in the reduction of blood glucose and serum cholesterol [6], and such beneficial health effects were shown to be correlated with characteristics of β-glucan, including the amount and molecular weight [7].

The potential use of oat β-glucans in the food and nutrition industry has been dependent on their physical properties, including rheological and hydrocolloid behavior. These physical properties were due to molecular features, including molecular weight, weight distribution and linkage patterns of cellulosic oligomers in the glucan chains, as well as the its concentration [8]. It was reported that the β-glucans with molecular weights of less than 100,000 formed a gel faster than those with a higher molecular weight [9]. In addition, the preparation method of gelation was also important [8]. Therefore, what is innovative in this work is that investigation of β-glucan gels properties with varying molecular weight and its distribution, and screening the optimal parameters in forming gels.

Food gels can be induced by heat treatment [10], acid [11], Ca^2+^ [12], enzyme [13], etc. Compared with other traditional methods, high pressure process (HPP), a nonthermal technology, can prepare food gels with unique properties and structures [14–18], which has led to its widespread used in food gel systems in recent years. In our previous work, the effects of high pressure treatments on gelation of β- lactoglobulin has been studied systematically [18], and the results revealed that high pressure treatment could change protein conformation and influence denaturation, aggregation and gelation, which could be used to develop novel functional foods. As the molecules responsible for the gelation are typically proteins or polysaccharides, the β-glucan gels, which should be met the requirements in the food industry by taking advantage of HPP, investigated for the first time based on the previously work. To take the lead in solving application limitation of β-glucan gels with different MWs, the current study focused on the β-glucan mixed gels with low and high MWs under high pressure, and explored mixed gel properties and formation mechanism, which were the unique view different from our previous study.

## Materials and methods

### Materials

The two kinds of oat β-glucan, OG (purity >85%) with a molecular weight of 2.0×10^5^, LOG (purity >85%) with a molecular weight of 1.0×10^5^, were purchased from Guangzhou Zhongkang food co. LTD (Guangdong, China). All other chemicals used were of analytical grade.

### Solution preparation

OG and LOG were dissolved in deionized water with different concentrations from 5% (w/v) to 15% (w/v) at varying mass ratios of 0:100, 25:75, 50:50, 75:25, 100:0(w/w). All the samples were magnetically stirred at 60 °C for 4 h to full dissolution and then stored at 25 °C until further use.

### High-pressure treatment

High-pressure operation was carried out with HPP L2-700/1 ultra-high pressure (Tianjin Huatai Senmiao Biotechnology and Technique Co. Ltd, Tianjin, China). Based on the operation procedure described in the previous study [18], oat β-glucan (mixed) solutions were subjected to high-pressure at 200 MPa, 400 MPa and 500 MPa for half hour at 25 °C, and the equipment pressure was then decreased to 0.1 MPa. A non-treated samples at 0.1 MPa were used as controls. After the operation, all the samples stored at 4 °C for 24 h until measuring.

### Rheological measurements

Rheological measurements of β-glucan gels were carried out by the dynamic oscillatory shear tests using a controlled stress rheometer AR-1500ex (TA Instruments, Delaware, USA) with a parallel plate geometry (40 mm diameter, 5 mm gap) at 25 °C controlled by the precise circulating water system. According to the previous study [18], the operation carried out in two steps.

First, the strain sweep test was performed to determine the linear viscoelastic region (LVR). The operation was carried out from 0.01 to 100% at a constant temperature of 25 °C and a constant frequency of 1 Hz. Second, the frequency sweep test was selected based on the LVR, and the angular frequency (ω) was set from 0.1 rad/s to 100 rad/s under a shear strain of 1%.

The values of the storage modulus (G′) and the loss modulus (G″) of the gels in the above two tests were obtained to analyze.

### Texture profile analysis

Textural profile analysis of the β-glucan gels was assessed using the TA-XT Plus Texture Analyzer (Stable Micro Systems, Godalming, UK). The operation was according to the method described by Li et al. with the minor modification [18]. Prior to measuring, each gel was cut into a cylindrical size (10 mm heightand 25 mm diameter) and equilibrated for 2 h at 25 °C. Then, the cylindrical gels were placed vertically and subjected to compression with a cylindrical probe (P/0.25) to a deformation of 30% using a 50 mm/min test speed and a trigger force of 0.4 N. All the measurements were undertaken at 25 °C and repeated six times. Hardness, cohesiveness, springiness and chewiness were used for evaluating the textural properties.

### Field emission scanning electron microscopy (FE-SEM)

The morphology of freeze-dried β-glucan mixed gels was imaged by a field emission scanning electron microscopy (FE-SEM, JSM-6701F, JEOL, Japan) at an accelerating voltage of 5.0 kV. Before observation, the sample were coated with a gold layer.

### Particle size measurements

The particle size of β-glucan (mixed) solutions was determined using a dynamic light scattering (DLS) (Nano-ZS90, Malvern Instruments, Worcestershire, U.K.). Prior to the test, the samples were diluted 1/500 (v/v) in distilled water and transferred to the cell. After the cell was placed on the equipment for equilibration for 2 min, dynamic light began to backscatter, and data were then collected for at least 10 sequential readings. The results of the particle size were expressed as cumulant mean diameter (size, nm). All the measurements were performed in triplicate at 25 °C.

### Turbidity measurements

Nephelometry experiments were carried out with a HACH 2100N Laboratory Turbidimeter (Loveland, TX, USA). The β-glucan (mixed) solutions were diluted 50 times in distilled water. All the experiments repeated three times at 25 °C.

### Molecular force of the β-glucan gel

The mixed solution (LOG: OG = 50:50, w/w) with a total concentration of 12% was prepared, then, 0.5, 1.0, 1.5, 2.0, 2.5 and 3.0 mol/L NaCl, urea and propylene glycol were added to the mixed solutions. All the samples were then subjected to 400 MPa for 30 min. The samples were stored at 4 °C overnight to carry out the textural and rheological properties tests.

### Fourier transform infrared (FTIR) spectroscopy

The infrared spectra of the samples were measured with a Spectrum 100 Fourier transform spectrophotometer (PerkinElmer, U.K.) in the 500–4000 cm^-1^ range, with a resolution of 4 cm^-1^. Pure KBr powder was used as a baseline, and then, 2.0 mg freeze-dried gels were mixed with 200 mg KBr and tableted into pellet. The data were processed by Omnic v8.0 (Thermo Nicolet, USA).

### Statistical analysis

The results were given as mean ±standard deviation and the means were obtained on three different determinations. ANOVA was used to compare the means, and Duncan multiple range test was used to determine the significance (p<0.05) of the difference by SPSS 18.0 (SPSS Inc., Chicago, USA).

## Results and discussion

### The appearance of the β-glucan gels

**Fig 1:**
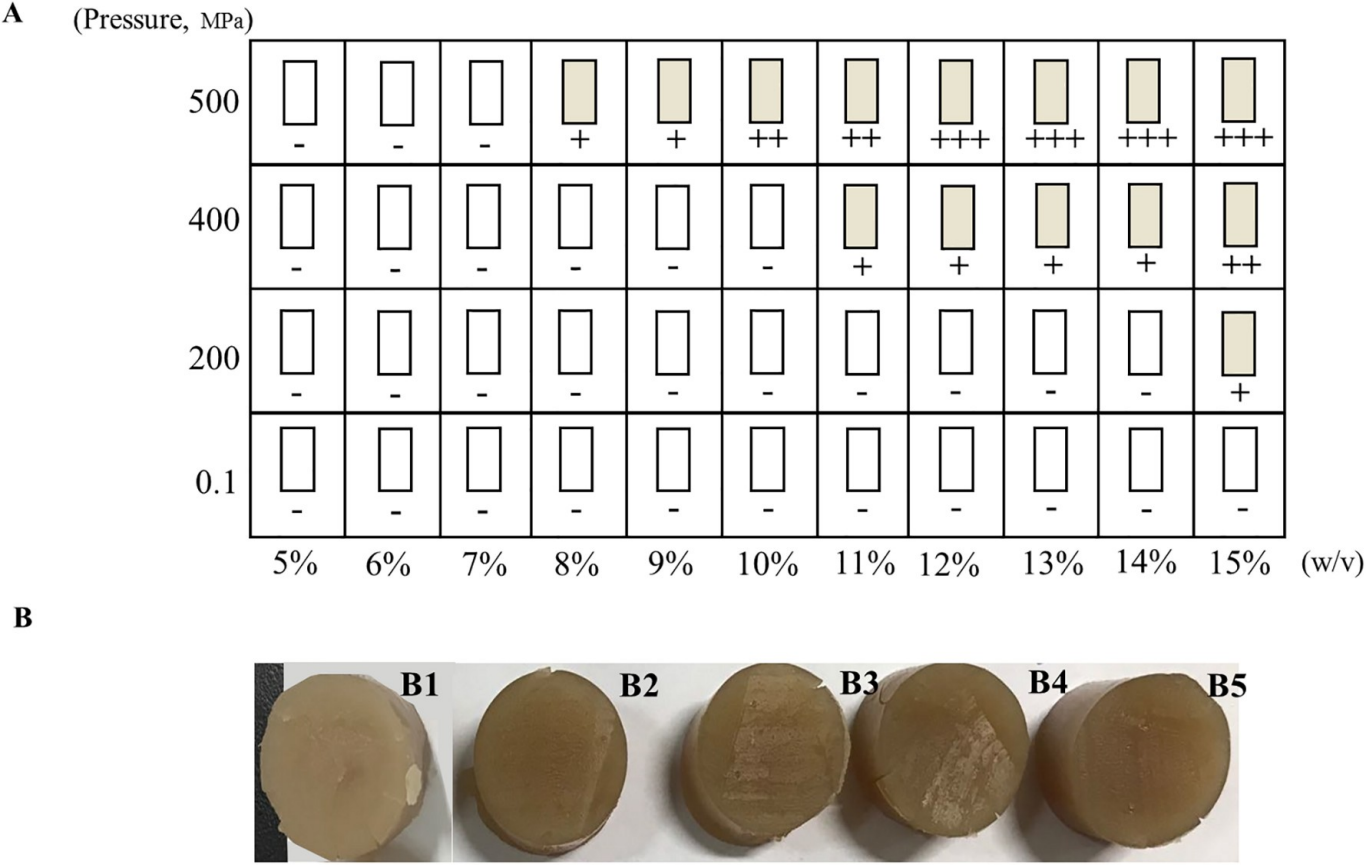
The states of β-Glucan (OG) gels prepared with varying concentrations under different pressures. (A) The states of β-Glucan (OG) gels. The solution state designated with a white rectangle, the gel state designated with a yellow rectangle, "-" means no gel; "+" means that the soft gel with coarser and less-compacted apparent; "++" means glossier and smoother gel; "+++" means that the gel with much smoother and more compact. (B) The photograph of β-Glucan (OG) gels with varying concentrations under 400MPa. B1-B5, total concentrations from 11% (w/v) to 15%(w/v).

Fig 1 shows the physical states of β-glucan (OG) gels under varying concentrations and pressures. The effects of different concentrations and pressures on gel formation were obvious. The gel formation didn’t been observed without HPP treatment, which was in agreement with another report [19]. The results indicated that a gel could form under relatively high pressure at a certain β-glucan concentration.

Under low pressure (200 MPa), the gel could form at the high concentration of 15% (w/v), while, with the rise of pressure from 400 to 500 MPa, the concentrations of forming gels decreased from 11% to 8%. In addition, the β-glucan gels with different concentrations under the same pressures showed different apparent states. With an increase in concentration, the appearance of the gel changed from a coarse state to a smooth state. The gel formed under 500 MPa with 15% showed a more compact state. A similar phenomenon was observed in Fig 1B. The gel formed at 400 MPa with 11% β-glucan showed the less-compacted appearance, even a little residue was observed, which could be related to the soft-gel. It was proved that β-glucan was in solution as irregular linear groups, which formed a viscoelastic system, and the gel formation was correlate with the specific concentration [20].

### Rheological properties of the β-glucan gels

#### The strain sweep

**Fig 2:**
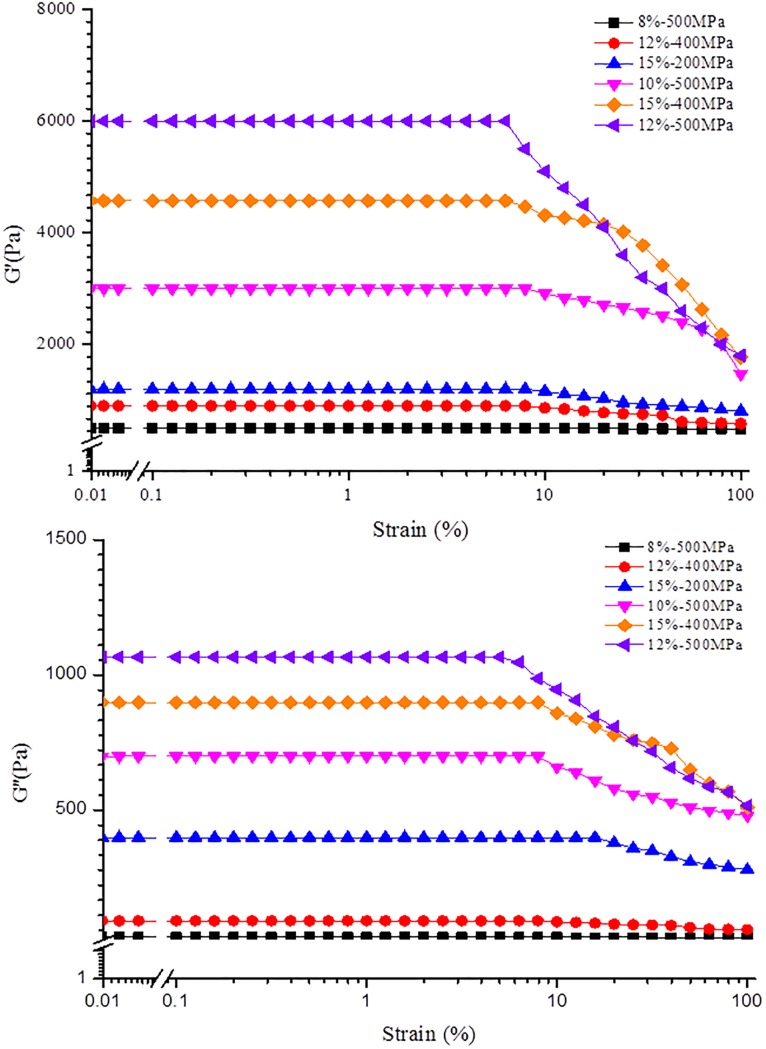
The strain sweep curves (G′ and G″) of β-glucan (OG) prepared with different concentrations at different pressures.

Fig 2 shows the strain sweep curves of β-glucan gels under different pressures and concentrations. It was obvious that the curves divided into two regions: the characteristic of the first region was a constant G′ value, which was called the certain strain range (LVE); the second region, called the nonlinear viscoelastic region, showed a sharp decrease in the G′ value with increasing strain. The results in Fig 2 indicated that β-glucan gels induced by HPP belong to macromolecular gels due to their LVE more than 1% [21].

The critical strain value γ_c_ was defined as the strain when G′ decreased to 95% of the initial value (G_0_) [22]. The γ_c_ of the gel decreased with increasing β-glucan concentration. The G′ of 8% (w/v) β-glucan gels at 500 MPa remained at a constant level in the strain range between 0.01 and 100%. In addition, the γ_c_ of 12% (w/v) β-glucan gels formed under 500 MPa was 9.7%, which indicated that the intensity of the gel prepared at high pressure was related to β-glucan concentration. It was observed that the G′ increased with the increasing β-glucan concentration when keeping the pressure constant, which was in accordance with another report [20]. Similarly, with increasing pressure, the G′ increased at the same concentration, which indicated that higher pressure could lead to an increase in the strength of β-glucan gels.

#### The frequency sweep

**Fig 3:**
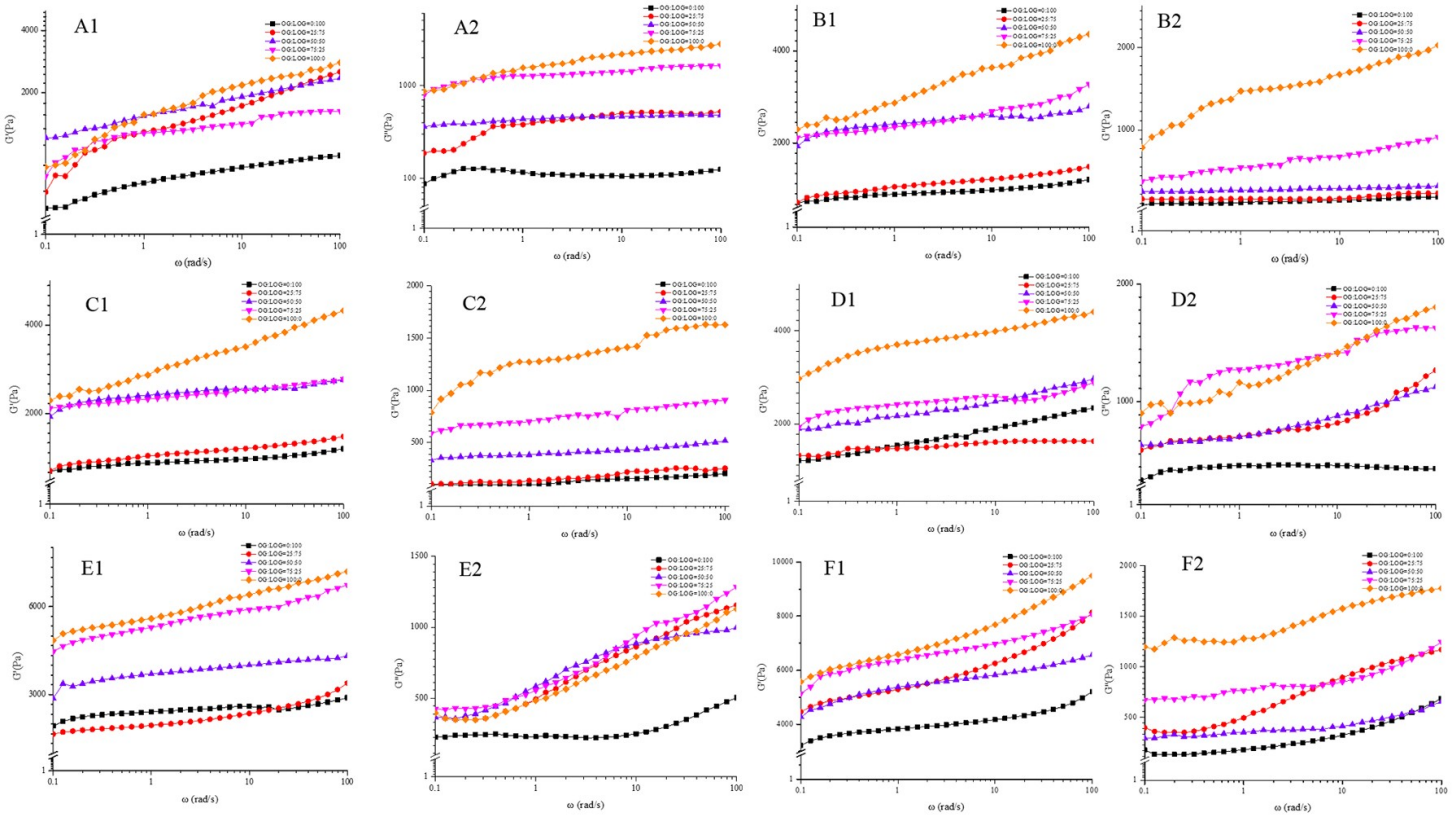
The frequency sweep curves (G′ and G″) of β-glucan mixed gels prepared at different pressure with varying total concentrations and ratios. (A) Total concentration of 8% under 500MPa. (B) Total concentration of 15% under 200MPa. (C) Total concentration of 12% under 400MPa. (D) Total concentration of 10% under 500MPa. (E) Total concentration of 15% under 400MPa. (F) Total concentration of 12% under 500MPa.

The effects of concentrations, pressures and ratios on the rheological properties of the β-glucan mixed gel are shown in Fig 3. The β-glucan concentration showed an obvious effect on the gel formation, and gelation occurred above the critical value of the β-glucan concentration. As seen in Fig 1, it was found that the lowest β-glucan (OG) concentration forming gels at 400 MPa was 11% (w/v) and 8% (w/v) at 500 MPa. Additionally, the ratio between OG and LOG played an important role in the rheological properties of β-glucan mixed gel.

The typical gel should form when the value of G′ is much larger than the G″ value, both G′ and G″ are rarely dependent on the frequency in the range from 10^−2^ rad/s to 10^2^ rad/s, and the G′ curve is parallel with the G″ curve [23,24]. It was seen in Fig 3 that the value of G′ was larger than that of the G″ under all the treatment conditions, while the mixed gels prepared with ratios of 75:25 and 50:50 were the typical gels (Fig 3B, 3C and 3D). In addition, a true elastic gel network can form when G′ is at least 1 order of magnitude greater than G″ and each value is rarely dependent on the frequency [25]. Therefore, the mixed gel induced at 200 MPa with 15% β-glucan and 400 MPa with 12% β-glucan formed the typical gels with a true elastic gel network.

It was reported that the molecular weight also played an important role in the gelation potential of cereal β-glucan [25]. The values of G′ in 100% and 50% LOG gels in Fig 3B and 3C were less angular frequency dependent, suggesting that the LOG contributed to a more elastic gel structure, which was explained by the fewer entanglements of low-MW β-glucan solutions leading to the lower apparent viscosity [26]. The ratio of 50:50 mixed gels showed a higher level of G′ than that of the β-glucan gel (OG), and the phenomenon was consistent at all different pressures and concentrations, which showed their stable network structure. This finding agreed with other research. It was speculated that the lower-MW β-glucan might serve as a linker molecule of the high-MW β-glucan [26].

We also observed an unparallel curve between G′ and G″ at a ratio of 75:25 and 100:0 in Fig 3D, 3E and 3F, which indicated the gels formed with these ratios could not belong with the typical gel. In fact, with the rise of the total β-glucan concentration, more OG was be added, which led to a large number of entanglements encountered. More entanglements could constrain the mobility of β-glucan, block the rearrangement into larger aggregated structures, which established the gel network. Therefore, addition the high-MW β-glucan contributed to a negative effect on the intermolecular interactions and rearrangement of strands into aggregated networks [27].

### Textural property of β-glucan gels

**Fig 4:**
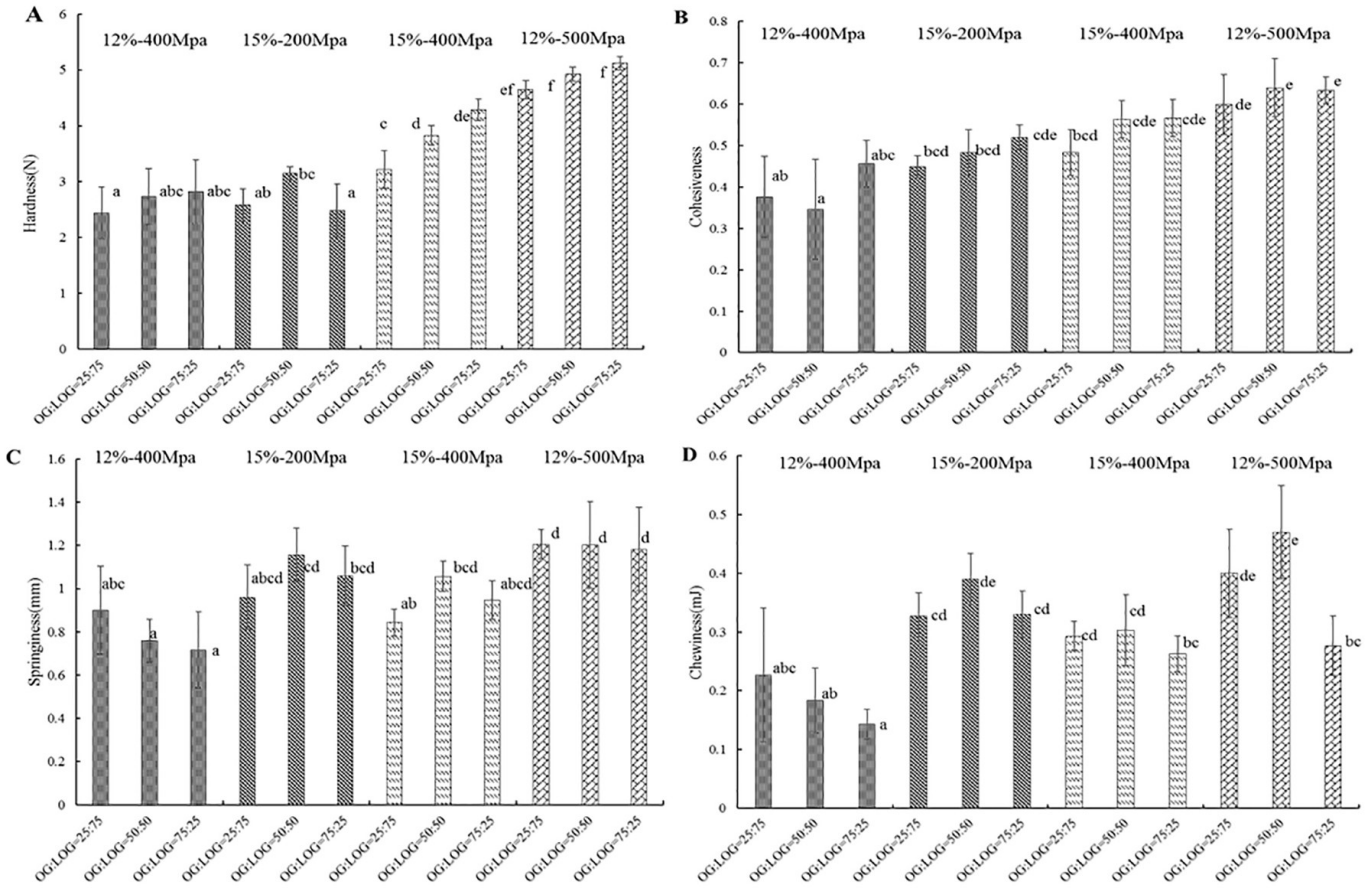
Textural property of the β-glucan mixed gels prepared at different pressure with varying total concentrations and ratios. (A) The hardness of the β-glucan mixed gels. (B) The cohesiveness of the β-glucan mixed gels. (C) The springiness of the β-glucan mixed gels. (D) the chewiness of the β-glucan mixed gels. Different superscript letters indicate significant differences in the same graph (*p* < 0.05).

Texture is an important property that determines the organoleptic quality of gels. The effects of pressures, concentrations and ratios on the textural properties of β-glucan mixed gels are shown in Fig 4. It can be seen that the concentration, ratio and pressure had obvious effects on the textural properties of gels. With increased the total concentrations of β-glucan from 12% to 14% under 400 MPa, the hardness and chewiness of the gels increased (*p*<0.05), which was consistent with the change of G′ levels, while the cohesiveness and springiness didn’t show a significant change.

The gels formed with a ratio of 50:50 and 75:25 showed a higher hardness than those with a ratio of 25:75. A similar finding was reported by other researches [26,28]. The β-glucan gels with high-MW formed a denser network, while those with the low-MW β-glucan with a low viscosity could move and set up junction zones more easily [29]. In addition, the increasing pressure led to large values of hardness, cohesiveness, springiness and chewiness of mixed gels (*p*<0.05). A previous report found that the pressure above 400 MPa could lead to forming starch gels [30].

### FESEM images of the β-glucan gels

**Fig 5:**
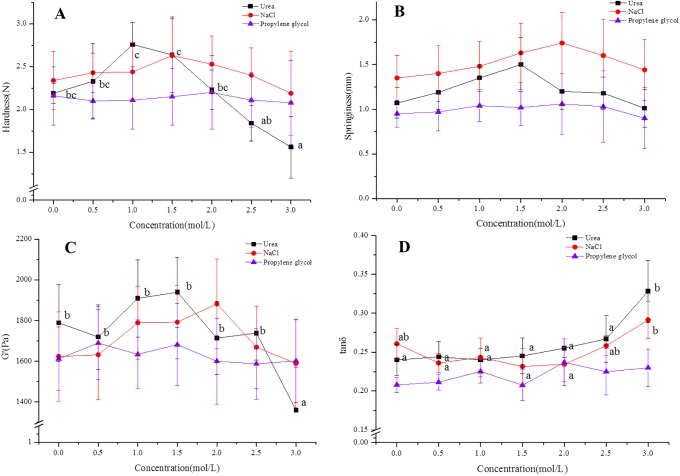
FESEM images of the β-glucan (mixed) gel prepared at different pressure with varying total concentrations and ratios. (A) 12%-0.1MPa, OG:LOG = 0:100. (B) 15%-200 MPa, OG:LOG = 50:50. (C) 12%-400MPa, OG:LOG = 50:50. (D) 15%-400MPa, OG:LOG = 50:50. (E) 12%-500MPa, OG:LOG = 50:50. (F) 12%-0.1MPa, OG:LOG = 100:0. The red arrows represent the holes.

The apparent structures of the β-glucan gels prepared under different conditions are shown in different images (Fig 5). The native β-glucan gel (LOG) showed a number of fragments, which indicated a failure informing a gel network (Fig 5A), while the gels with a number of pores are shown in Fig 5B and 5C, and a smooth surface without certain pores is observed in Fig 5D and 5E. Low-MW β-glucan, binding sufficient water, formed an unstable gel network, which was easily collapsed. Meanwhile, gels with a ratio of 50:50 formed the harder, more elastic gels without many pores, which was attribute to high-MW β-glucan forming the elastic structure and the low-MW β-glucan acting as linker molecules in the high-MW β-glucan [26]. The increasing in concentration and pressure led to the compact structure, this phenomenon followed the measurement results of hardness, springiness and chewiness. These findings might be attributed to the intermolecular and intramolecular forces of different β-glucan molecules [31].

### The turbidity and particle size measurement of the β-glucan solution

**Fig 6:**
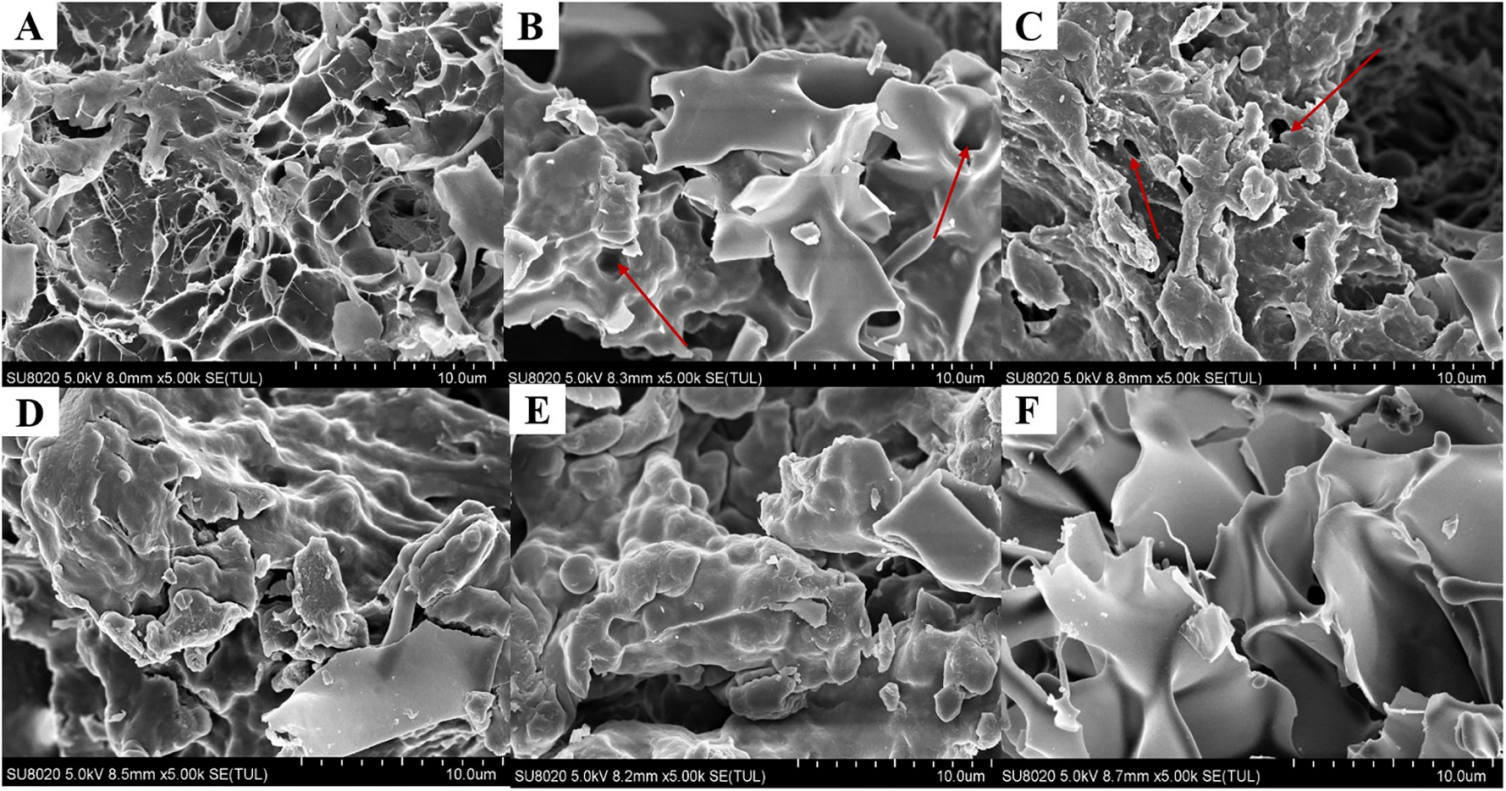
The turbidity and particle size of the β-glucan mixed solutions with different concentrations and ratios under different pressures. (A) The average size of the β-glucan mixed solutions. (B) The turbidity of the β-glucan mixed solutions. Different superscript letters indicate significant differences in the same graph (*p* < 0.05).

Fig 6 shows the turbidity and particle size of β-glucan mixed solutions under different conditions. Compared to the high-pressure treated samples, the untreated sample showed slightly larger particles. Under 200 MPa, the particles size in the β-glucan solution remained the same as the untreated samples in all the ratios, suggesting that relatively low pressure could not reduce the particle sizes. At 400 MPa, the particle sizes of β-glucan mixed solution (OG:LOG = 50:50) abruptly decreased (*p*<0.05), the finding corresponded with Ahmed’s report [19]. The sudden increase in pressure might create cavitation, and the fluctuating collapse of bubbles in the fluid might shock waves, which may lead to the disruption of particles [32]. It was reported that increasing pressure could reduce the particle size of different polysaccharides, such as starch and pectin [33,34]. In addition, the OG/LOG ratio had a significant effect on the β-glucan solution particle size. The particle sizes of the mixed solutions under high pressure were smaller than that of the β-glucan single solution, which might be attributed to the intermolecular and intramolecular forces between different β-glucan molecules and water. β-Glucan solutions with the low MW showing a low apparent viscosity could increase their mobility, which were easily formed into junction zones through hydrogen bonds [26].

In Fig 6B, it was observed that the turbidity of the β-glucan solution without HPP was slightly higher than that of the treated solution under 200 MPa, while increasing the pressure to the 400MPa could elevate the turbidity of the β-glucan solution. A Similar phenomenon was also observed in Chens’ reports [35,36]. It might indicate that certain pressures could induce the aggregation of β-glucan. The previous study had approved that the pressures up to 250 MPa could lead to the actomyosin formed aggregates and lost the arrowhead structure [37]. The aggregation of protein molecules was positively correlative with the turbidity change of protein solution [38]. Chen reported myosin-K-carrageenan solution had undergone unfolding and aggregation under 200–400 MPa [36].

### The molecular forces of the formation of the β-glucan mixed gels

**Fig 7:**
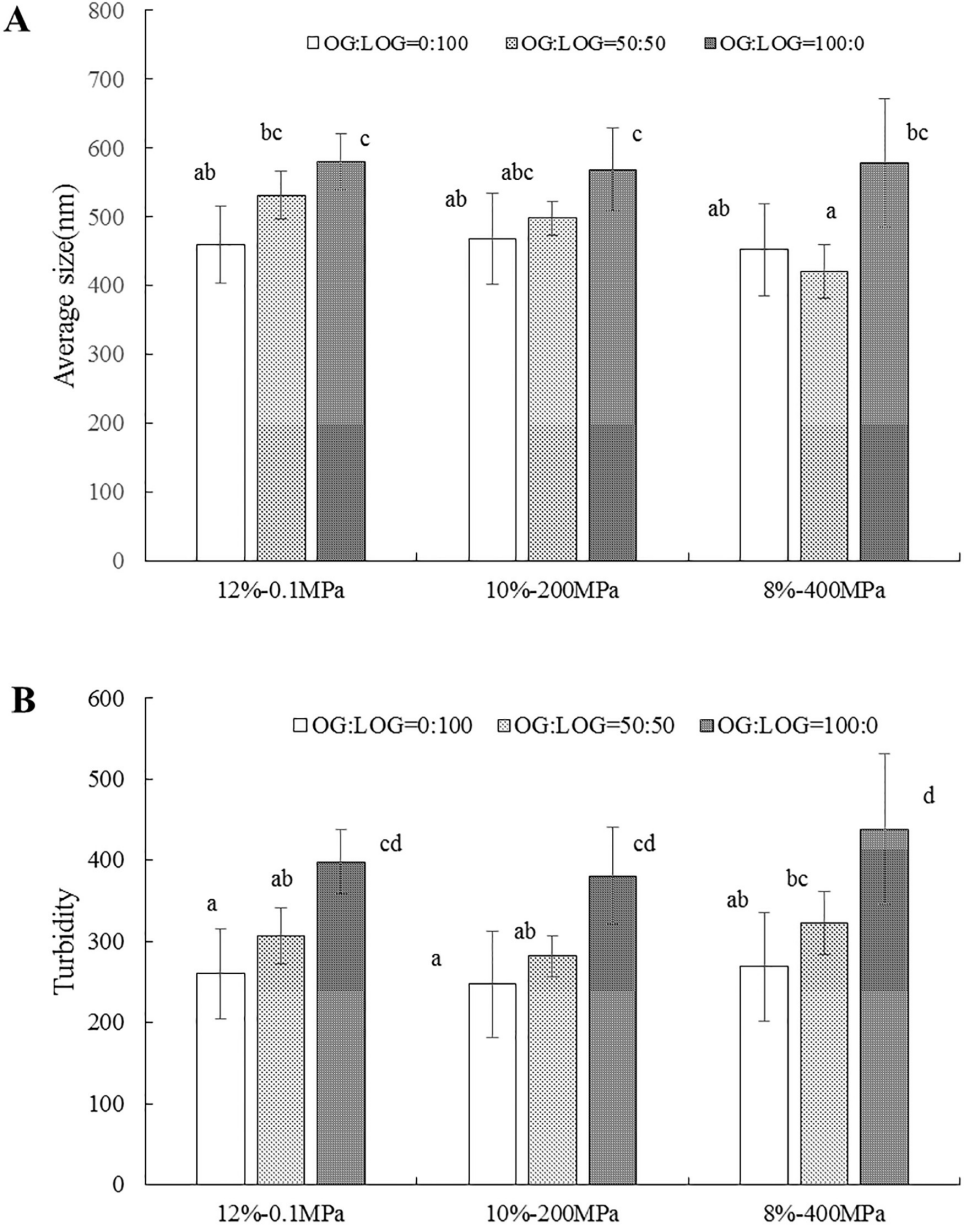
The effects of NaCl, urea and propylene glycol on hardness and springiness of the β-glucan mixed gels. (A) The hardness of the β-glucan mixed gels. (B) The springiness of the β-glucan mixed gels. (C) The G′ value of the β-glucan mixed gels. (D) The tan δ value of the β-glucan mixed gels. Different superscript letters indicate significant differences in the same graph (*p* < 0.05).

Fig 7 shows that the relationship between the rheological and textural properties of the mixed gels with the different concentrations of NaCl, urea and propylene glycol, which can reveal the strength of the three kinds of interaction including hydrogen bonding, hydrophobic and electrostatic interaction. High concentrations of NaCl can screen charge effects and reduce electrostatic interactions. Urea can hinder the formation of hydrogen bonds by affecting the structure of water molecules. Propylene glycol can affect water structure, might disrupt hydrophobic forces and promote hydrogen and electrostatic bonds [39]. The formation of β-glucan mixed gels induced by HPP might be the result of synergistic effects of Van der Waals forces, hydrogen bonding, hydrophobic, and electrostatic interaction. The intermolecular Van der Waals forces were smaller and can be neglected due to β-glucan belonging to the biopolymer materials category.

As seen in Fig 7A and 7B, with the rise of urea concentration, the hardness and springiness of β-glucan mixed gel increased initially and then decreased, of which a change in the hardness with increasing urea concentration was obvious (*p*<0.05). These results suggested that hydrogen bonding and electrostatic interaction played the main role in the mixed gels induced by HPP. This finding agrees with a previous study [26].

Although urea could impair intramolecular and intermolecular hydrogen bonds, low concentration of urea could unfold the β-glucan chain, which might lead the β-glucan with relatively high concentrations to rearranging and forming the network structure of the gel, showing an increase in hardness and elasticity [27]. With increasing urea concentrations, hydrogen bonds between urea molecules formed, at the same time, strong competitive hydration might lead to random aggregation, which could reduce the formation of the network, which would manifest as a decrease in hardness and elasticity[40], while the hardness and springiness were kept at a constant level with addition in different concentrations of propylene glycol, which indicated the hydrophobic interactions were not important in forming the β-glucan mixed gel induced by HPP.

Fig 7C and 7D show the G′ and tan δ values at 1 Hz of mixed gels (OG:LOG = 50:50) with 12% β-glucan under 400 MPa. With the rise of urea and NaCl concentrations, the G′ levels of β-glucan mixed gels increased initially and then decreased, while tan δ values had an increasing tendency. The value of tan δ, defined as the energy lost to the energy stored (G″/ G′) in a system, indicated the solid properties (tan δ <1) [20]. The tendency of tan δ to change was consistent with that of less elastic gels showing higher tan δ values [27], this might occur because the higher concentration of NaCl could shield the charge effect and reduce the electrostatic interaction between β-glucan molecules. On the other hand, high concentrations of NaCl brake the hydration bond and hydrogen bond, change the formation of the water around the gel-forming area and destroy the stability of the gel network structure, thus, reducing the gel strength [41].

### The FTIR of the β-glucan mixed gels

**Fig 8:**
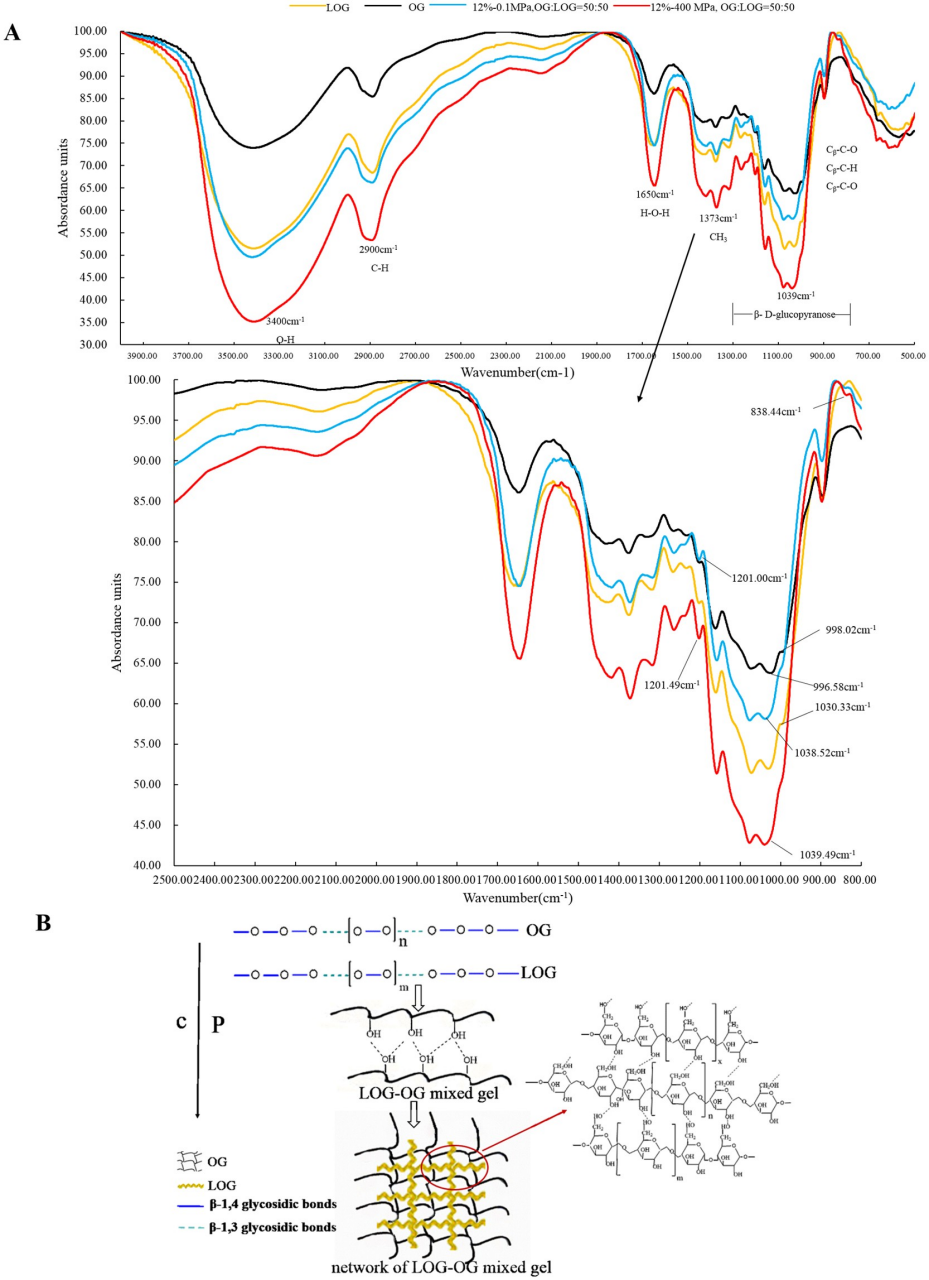
The possible formation mechanism of mixed gel between LOG and OG. (A) The FTIR spectroscopy of the β-glucan mixed gels. (B) The possible formation mechanism of the mixed gel.

To investigate the structure of β-glucan mixed gels induced by HPP, FTIR spectroscopic analysis of β-glucan mixed gels (OG:LOG = 50:50, w/w) under 400 MPa was performed, and the results are shown in Fig 8A.

In the functional group region, there are significant absorptions at approximately 3400 cm^-1^ and 2900 cm^-1^, which correspond to the stretching absorption bands of poly -OH and C-H. The absorptions peaks around 1600 cm^-1^ are attributed to the stretching vibration of water. The peak at 1418 cm^-1^ is attributed to the methylene bending vibration peak [42,43]. The peaks around 1373 cm^-1^ are attributed to the symmetrical angular vibration peak of methyl. The peak at approximately 1300 cm^-1^ belongs to the bending vibration peak of C-O-H bond. The absorption peak of 1200–1000 cm^-1^ belongs to the stretching vibration peak of C-O-C, indicating that the monosaccharides of β-glucan are connected by C-O-H, 1158 and 1076 cm^-1^ peaks belong to the stretching vibration peak of C-O-C, and the absorption peak around 1000 cm^-1^ is the characteristic peak of the variable angle vibration of C-H in β-D-pyranose, indicating that the molecule contains the β-D-pyranose ring, and the molecule is connected by the β-glycoside bond [44].

It was obvious that there were no new absorption peaks between the single β-glucan and mixed gel (without and with HPP), which indicated that the complex compounds basically did not change its primary structure. In addition, the spectra showed that there was no change in the selected bands undertaking HHP, which indicated that the high pressure left no significant changes in the core structure of β-glucan; these results agreed with those in a previous report [19]. Compared with the mixed gels and single β-glucan, the certain absorption peak intensity changed, namely, the absorption peak at approximately 3400 cm ^-1^, the absorption intensity order was observed as follows: mixed gel induced by HHP > mixed solution without HHP >single β-glucan, which indicated intermolecular hydrogen bonds played an important contribution to the formation of the mixed gels induced by HPP, this conclusion was consistent with the finding seen in Fig 7. Compared with the stretching vibration peak of C-O-C around 1030 cm^-1^ in β-glucan (LOG), the peak showed the redshift (998 cm^-1^) in the mixed gel, which might indicate that the number of beta 1,3 and 1,4 bonds formed in the mixed gel and the single β-glucan (OG) changed. It can be seen in Fig 8A that the mixed gel had a weak absorption peak at 838.44 cm^-1^, and it is speculated that part of the a-glycoside bond was formed during the gel formation process [42]. Compared with the single β-glucan, the absorption peak of mixed gel at 1200 cm^-1^ was obvious, which was preliminarily concluded to be carbonyl stretching vibration, indicating that the connection of glycoside bonds might be changed during the gel formation process [45], which could speculate the possible formation mechanism of mixed gel between LOG and OG showed in Fig 8B with schematic.

### Conclusions

This research investigated the effects of different β-glucan concentrations and pressure on the gelation of β-glucan mixed gels with different MW β-glucan induced by HPP. The characterization and formation mechanism of oat β-glucan mixed gels with different MW induced by high pressure was explored. The rheological and textural properties of β-glucan mixed gels increased with increasing of concentrations and pressures. A typical mixed gel with a true, compact and smoother elastic network formed with 12% β-glucan at the ratio of 50:50 under 400 MPa for 30 min. Based on the particle size, molecular forces and structural investigation, the finding showed that high pressure did not change the core molecular structure of β-glucan, and the hydrogen bonding and electrostatic interaction played a critical role during the formation of β-glucan mixed gels by HPP. This study manifested that high-pressure is an alternative technology for inducing β-glucan gel formation, and this application in modification of the food composition will addressed in a future study.
